# Sorafenib administered using a high-dose, pulsatile regimen in patients with advanced solid malignancies: a phase I exposure escalation study

**DOI:** 10.1007/s00280-020-04065-5

**Published:** 2020-04-09

**Authors:** L. H. Mammatas, A. S. Zandvliet, M. Rovithi, R. J. Honeywell, E. L. Swart, G. J. Peters, C. W. Menke-van der Houven van Oordt, H. M. W. Verheul

**Affiliations:** 1 Department of Medical Oncology, Cancer Center Amsterdam, Amsterdam UMC, VUmc University Medical Center Amsterdam, Amsterdam, The Netherlands; 2 Department of Clinical Pharmacology and Pharmacy, Cancer Center Amsterdam, Amsterdam UMC, VUmc University Medical Center Amsterdam, Amsterdam, The Netherlands; 3 Department of Biochemistry, Medical University of Gdansk, Gdansk, Poland; 4 Department of Medical Oncology, Radboud University Medical Center, Geert Grooteplein Zuid 8, Internal postal code 452, P.O. Box 9101, 6500 HB Nijmegen, The Netherlands

**Keywords:** Sorafenib, Phase I clinical trial, High dose, Pulsatile, Drug monitoring, Cola

## Abstract

**Background:**

(Pre)clinical evidence is accumulating that intermittent exposure to increased doses of protein kinase inhibitors may improve their treatment benefit. In this phase I trial, the safety of high-dose, pulsatile sorafenib was studied.

**Patients and methods:**

High-dose sorafenib was administered once weekly in exposure escalation cohorts according to a 3 + 3 design. Drug monitoring was performed in weeks 1–3 and doses were adjusted to achieve a predefined target plasma area under the curve (AUC)(0–12 h). The effect of low gastric pH on improving sorafenib exposure was investigated by intake of the acidic beverage cola.

**Results:**

Seventeen patients with advanced malignancies without standard treatment options were included. Once weekly, high-dose sorafenib exposure was escalated up to a target AUC(0–12 h) of 125–150 mg/L/h, achieving a twofold higher *C*_max_ compared to standard continuous dosing. Dose-limiting toxicity was observed in three patients: grade 3 duodenal perforation (2800 mg sorafenib), grade 5 multiorgan failure (2800 mg sorafenib) and grade 5 biliary tract perforation (3600 mg sorafenib). The mean difference between observed and target AUC(0–12 h) was 45% (SD ± 56%) in week 1 using a fixed starting dose of sorafenib compared to 2% (SD ± 32%) in week 3 as a result of drug monitoring (*P* = 0.06). Dissolving sorafenib in cola, instead of water, did not improve sorafenib exposure. Clinical benefit with stable disease as the best response was observed in two patients.

**Conclusion:**

Treatment with high-dose, once weekly sorafenib administration resulted in dose-limiting toxicity precluding dose escalation above the exposure cohort of 125–150 mg/L/h. Drug monitoring was a successful strategy to pursue a target exposure.

**Electronic supplementary material:**

The online version of this article (10.1007/s00280-020-04065-5) contains supplementary material, which is available to authorized users.

## Introduction

Sorafenib is an oral multikinase inhibitor, originally developed as an inhibitor of RAF kinases [1]. Besides activity against C-RAF, B-RAF and mutant B-RAF, it also inhibits vascular endothelial growth factor receptors 1, 2 and 3, platelet-derived growth factor receptor β, FMS-like tyrosine kinase 3, c-Kit protein and RET receptor tyrosine kinase at low concentrations [2]. At high intracellular concentrations, sorafenib has affinity for multiple other kinases [3]. Sorafenib is currently approved for the treatment of renal cell carcinoma, hepatocellular carcinoma and iodine refractory thyroid cancer at a standard fixed dose of 400 mg twice daily in a continuous schedule [4–7]. However, at this standard fixed dose large interpatient variability in drug exposure was demonstrated after both single and multiple doses [8].

Increased sorafenib exposure is associated with improved efficacy [9, 10]. Dose escalation of sorafenib to 600 mg twice daily after failure of standard dosing in patients with progressive renal cell carcinoma resulted in tumor reduction in 42% of the patients [9]. In addition, intrapatient dose escalation in patients without substantial toxicity showed that a higher area under the concentration–time curve (AUC_max_ > 100 mg/L/h) of sorafenib was associated with longer progression-free survival (PFS) [10]. Unfortunately, toxicity limits further dose escalation of the continuous schedule [8].

An alternative approach to achieve high exposure, with less toxicity, may be high-dose, pulsatile administration of protein kinase inhibitors [11, 12]. Recently, we showed promising preclinical and clinical benefit of an alternative high-dose treatment regimen of sunitinib [13]. Also, promising preclinical results for high-dose sorafenib were reported. In mice bearing 789-O renal cell carcinoma xenografts, such a schedule exhibited increased reduction of tumor perfusion and microvessel density as well as slower tumor growth in comparison to continuous conventional dosing [14].

An important challenge for optimizing high-dose sorafenib administration is the amount of drug absorption in the gastrointestinal tract. Sorafenib absorption is saturable > 800 mg/day in a daily continuous schedule. However, a moderate fat meal (in comparison to a high fat meal of ≥ 50% fat) and multiple divided doses per day have been shown to improve the absorption of sorafenib by 30% and 50%, respectively [15, 16]. The influence of gastric pH on the absorption of sorafenib is less clear, although the solubility of sorafenib increases with decreasing pH and ranges from 0.034 mg/100 mL at pH 1.0 to 0.013 mg/100 mL at pH 4.5 [17–19]. Thus, the administration of an acidic beverage such as classic cola, with a pH of 2.5, could potentially improve sorafenib absorption and bioavailability [20, 21].

Based on these considerations, we initiated a clinical phase I study with high-dose, pulsatile sorafenib. Weekly pulses of high sorafenib exposure over a 12-h window [AUC(0–12 h)] were pursued in an attempt to improve clinical efficacy. To overcome saturation of absorption, we applied dose fractioning (portions of 200–400 mg administered at 2 h intervals), a standardized moderate fat diet, and investigated the effect of cola on sorafenib bioavailability. Sorafenib exposure was determined during 12 h following ingestion of the last dose fraction. Finally, administration of the same dose was anticipated to result in large variability in sorafenib plasma AUC(0–12 h) per patient [8]. Therefore, drug monitoring was performed during weeks 1–3 to titrate the patients’ individual dose based on the sorafenib plasma AUC(0–12 h) according to exposure escalation cohorts [22].

## Patients and methods

### Patient selection

Patients were eligible if they had a pathologically confirmed solid malignancy refractory to standard therapy or if no standard therapy existed for them. Patients had to be ≥ 18 years of age with an Eastern Cooperative Oncology Group Performance of ≤ 1. Required laboratory values at entry included hemoglobin ≥ 5.6 mmol/L, absolute neutrophil count ≥ 1.5 × 10^9^/l, platelet count ≥ 100 × 10^9^/l, total bilirubin ≤ 1.5 times the upper limit of normal (ULN), ALT and AST ≤ 2.5 × ULN (in case of liver metastases: ≤ 5 times ULN), serum creatinine ≤ 1.5 × ULN or creatinine clearance ≥ 50 ml/min (based on MDRD), albumin> 25 g/L, PT-INR/PTT < 1.5 × ULN (unless coumarin derivatives were used), and activated partial thromboplastin time < 1.25 × ULN.

The main exclusion criteria were other anticancer therapies within 4 weeks (6 weeks for nitrosoureas and mitomycin C); evidence of serious uncontrolled concomitant disease (such as cardiovascular disease, nervous system disease, pulmonary disease, gastrointestinal disorders or active bacterial, viral, fungal or mycobacterial infections); uncontrollable hypertension (> 160/95 mmHg); prior radiotherapy on the abdominal or thoracic area or on > 3 vertebrae; major surgery within 4 weeks; pregnancy or breast feeding. If applicable, patients were required to take contraceptive precautions while on the trial and for 6 months afterwards.

All patients gave written informed consent before study entry and the local medical ethics committee of the Amsterdam UMC, location VUmc (Medisch Ethische Toetsingscommissie VUmc), approved the study. The study (NCT02636426) was conducted in accordance with the Declaration of Helsinki and Good Clinical Practice guidelines.

### Study design and treatment plan

This single center phase I study was conducted at the Amsterdam UMC, location VUmc, the Netherlands. High-dose sorafenib was administered once weekly in exposure escalation cohorts that consisted of 3–6 patients using a standard 3 + 3 design. Drug monitoring was performed in weeks 1–3 and doses were adjusted a maximum of two times if necessary to achieve the predefined target plasma AUC(0–12 h) of the cohort. The starting exposure level was 25–50 mg/L/h, analogous to the continuous schedule [23], and was escalated in subsequent cohorts with increments of 25 mg/L/h.

The primary objective was to investigate the maximum tolerated plasma AUC(0–12 h) of high-dose, pulsatile sorafenib and its safety and tolerability. Secondary objectives were (1) the pharmacokinetic behavior, (2) the influence of cola on sorafenib exposure, (3) the feasibility of drug monitoring to achieve the target plasma AUC(0–12 h), and (4) preliminary evidence of improved anticancer activity with high-dose pulsatile sorafenib treatment of this alternative sorafenib treatment strategy.

The total weekly dose sorafenib was divided in portions of 200–400 mg and given every 2 h to prevent saturation of absorption and to result in a high plasma peak concentration at the end of all ingested portions. Each dose was dissolved in either a large glass of water or classic Coca-Cola (the Coca-Cola Company, Atlanta GA) (~ 240 ml). Furthermore, patients used a standard low-fat diet (± 14 g fats, 100 g proteins, 1800 kcal) with as well as between doses on the day of administration to optimize the absorption and bioavailability of sorafenib.

Patients continued study treatment until unacceptable toxicity, disease progression or the patient’s request to stop. Evaluable patients had to be treated for a minimum of 2 weeks or would otherwise be replaced by an additional patient.

### Safety assessment

Safety and tolerability assessments, including physical examination, ECG and blood hematology and chemistry, were performed weekly during the first 8 weeks and once every 4 weeks thereafter. Adverse events (AEs) were graded according to the National Cancer Institute Common Terminology Criteria for Adverse Events version 4.0.

Dose (/exposure)-limiting toxicity (DLT) was defined as any grade 3 toxicity that occurred within the first 6 weeks of treatment and possibly related to the study drug. The maximum tolerated exposure (MTE) was defined at the highest exposure level at which ≤ 33% of patients experienced DLTs.

### Tumor response measurements

Tumor response was assessed by computed tomography or magnetic resonance imaging at baseline and every 8 weeks thereafter using RECIST version 1.1 [24].

### Pharmacokinetic analysis

During the first 3 weeks of study treatment, blood samples for measurement of sorafenib and its active metabolite sorafenib N-oxide were taken prior to each dose (i.e. prior to each portion of the total weekly dose given every 2 h) and 1, 2, 3, 4, 8, 12 and 60–96 h after the last dose.

The plasma concentrations of sorafenib and sorafenib N-oxide were determined using a validated liquid chromatography-tandem mass spectrometry method [25]. The sorafenib and sorafenib N-oxide plasma AUC(0-12 h) were determined from the time of the last sorafenib tablet ingestion until 12 h afterwards with a non-compartmental method. Within 72 h after ingestion, the sorafenib AUC(0–12 h) was established by our department of Clinical Pharmacology and Pharmacy and if necessary the dose was adjusted accordingly to achieve the target AUC(0–12 h) of the exposure cohort. Per patient a maximum of two dose adjustments were permitted. In addition, the maximum concentration (C_max_) and time of maximum concentration (*T*_max_) were determined.

### Statistical considerations

Descriptive statistics were used to describe patient characteristics, treatment administration, safety, efficacy and pharmacokinetic data. Progression-free survival (PFS) was defined as the time from first treatment until progression of disease or death as a result of any cause. Fisher’s exact tests were used to assess correlations between exposure level or dose and frequency of AEs. A paired *t* test was used to investigate the effect of personalized dose titration on achieving the target sorafenib AUC(0–12 h). Last, a Mann–Whitney test was used to investigate the effect of cola on dose normalized (to a standard dose of 800 mg sorafenib) plasma sorafenib *C*_max_ and AUC(0–12 h) levels. Data were considered significant at *P* < 0.05.

## Results

### Patients and treatment

Seventeen patients with progressive metastatic malignancies were enrolled between November 2015 and December 2017. Patient characteristics are shown in Table 1. A total number of 114 weekly cycles of sorafenib were administered with a median of 7 cycles per patient (range 1–24).

**Table 1:** Patient characteristics

Characteristic	Number of patients, *N* = 17
Age (years) Median (range)	61 (26–74)
Gender Males/females	7/10
ECOG performance status 0/1	2/15
Tumor type Pancreas Bile duct Head and neck Esophagus Colorectal Kidney Liver (HCC) Melanoma Uterus Breast Sarcoma Metaplastic carcinoma	2 2 2 2 2 1 1 1 1 1 1 1
Prior treatment Surgery Radiotherapy Chemotherapy Protein kinase inhibitor	8 6 13 3 (1 patient prior used standard dose sorafenib)
Number of prior systemic regimens Median (range)	2 (0–6)

Patients were treated at target AUC(0–12 h) levels from 25–50 to 125–150 mg/L/h. Because the first exposure cohort [AUC(0–12 h) 25–50 mg/L/h] already resulted in higher sorafenib exposure than expected [median AUC(0–12 h) 71, range 61–103 mg/L/h], the second exposure cohort was set at 75–100 mg/L/h. In subsequent cohorts, target exposure levels were increased with steps of 25 mg/L/h.

Reasons for treatment discontinuation were disease progression (*N* = 11), treatment-related toxicity (*N* = 3), completion of the study protocol, i.e. treatment discontinuation because ≥ 33% of patients experienced DLTs in that cohort (*N* = 1), patient withdrawal (*N* = 1) and a pathological bone fracture (*N* = 1). The latter two patients were considered non-evaluable as described in the protocol, because they had only received 1 week of study treatment and were replaced.

### Safety

Adverse events which were at least possibly related to high-dose sorafenib are summarized in Table 2. Most common clinical toxicities were fatigue (67%), nausea (67%), vomiting (53%) and diarrhea (27%). These grade 1–2 toxicities typically started 1–2 days after sorafenib administration and were manageable with standard supportive care measures.

**Table 2:** Treatment-related adverse events

Toxicity	Grade	All cohorts (*N* = 15)	Cohort 1 (*N* = 3)	Cohort 2 (*N* = 6)	Cohort 3 (*N* = 3)	Cohort 4 (*N* = 3)	Dose < 2400 mg (*N* = 11)	Dose ≥ 2400 mg (*N* = 9)	Observed AUC < 100 mg/L/h (*N* = 14)	Observed AUC ≥ 100 mg/L/h (*N* = 11)
Fatigue	All	10 (67%)	1 (33%)	5 (83%)	3 (100%)	1 (33%)	4 (36%)	6 (67%)	3 (21%)	7 (64%)
	Grade ≥ 3	0	0	0	0	0	0	0	0	0
Nausea	All	10 (67%)	2 (67%)	4 (67%)	2 (67%)	2 (67%)	5 (45%)	5 (56%)	5 (36%)	5 (45%)
	Grade ≥ 3	0	0	0	0	0	0	0	0	0
Vomiting	All	8 (53%)	2 (67%)	3 (50%)	1 (33%)	2 (67%)	5 (45%)	3 (33%)	7 (50%)	1 (9%)
	Grade ≥ 3	0	0	0	0	0	0	0	0	0
Diarrhea	All	4 (27%)	1 (33%)	1 (17%)	1 (33%)	1 (33%)	2 (18%)	2 (22%)	2 (14%)	2 (18%)
	Grade ≥ 3	0	0	0	0	0	0	0	0	0
Anorexia	All	3 (20%)	0	2 (33%)	1 (33%)	0	0	3 (33%)	1 (7%)	2 (18%)
	Grade ≥ 3	0	0	0	0	0	0	0	0	0
Bloating	All	2 (13%)	0	1 (17%	0	1 (33%)	1 (9%)	1 (11%)	1 (7%)	1 (9%)
	Grade ≥ 3	0	0	0	0	0	0	0	0	0
Stomatitis	All	2 (13%)	0	1 (17%)	1 (33%)	0	0	2 (22%)	0	2 (18%)
	Grade ≥ 3	0	0	0	0	0	0	0	0	0
Perforation	All	2 (13%)	0	1 (17%)	0	1 (33%)	0	2 (22%)	1 (7%)	1 (11%)
	Grade ≥ 3	2 (13%)	0	1 (17%)	0	1 (33%)	0	2 (22%)	1 (7%)	1 (11%)
Multiorgan failure	All	1 (7%)	0	0	0	1 (33%)	0	1 (11%)	1 (7%)	0
	Grade ≥ 3	1 (7%)	0	0	0	1 (33%)	0	1 (11%)	1 (7%)	0
ALP increased	All	3 (20%)	0	0	1 (33%)	2 (67%)	0	3 (33%)	1 (7%)	2 (18%)
	Grade ≥ 3	2 (13%)	0	0	0	2 (67%)	0	2 (22%)	1 (7%)	1 (9%)
Bilirubin increased	All	2 (13%)	0	0	0	2 (67%)	0	2 (22%)	1 (7%)	1 (9%)
	Grade ≥ 3	0	0	0	0	0	0	0	0	0
GGT increased	All	1 (7%)	0	0	0	1 (33%)	0	1 (11%)	1 (7%)	0
	Grade ≥ 3	1 (7%)	0	0	0	1 (33%)	0	1 (11%)	1 (7%)	0

Serious adverse events were predominantly observed in the gastrointestinal tract. At the target exposure level of 75–100 mg/L/h, one patient developed grade 5 biliary tract perforation after three cycles of treatment (sorafenib dose was 3600 mg with an observed AUC(0–12 h) of 182 mg/L/h). The cohort was expanded to six evaluable patients, but no further DLT occurred. At the subsequent 100–125 mg/L/h target exposure level, three patients were treated without DLT. However, a DLT occurred in two out of three patients in the 125–150 mg/L/h cohort. One patient developed grade 3 duodenal perforation after two cycles of treatment (sorafenib dose was 2800 mg with an observed AUC(0–12 h) of 54 mg/L/h). The other patient suffered from grade 5 multiorgan failure after two cycles of treatment (sorafenib dose was 2800 mg with an observed AUC(0–12 h) of 47 mg/L/h) and died. At that point, the phase I study was preliminary terminated because these serious toxicities precluded further dose escalation and investigation of a potential benefit of a high-dose, pulsatile approach.

Overall, the frequency and rate of grade ≥ 3 AEs increased with a higher dose of sorafenib (*P* = 0.003 and *P* = 0.008, respectively) but did not increase with higher exposure (*P* = 0.43 and *P* = 0.70, respectively). Grade ≥ 3 AEs developed from doses ≥ 2800 mg/week. Dose interruptions were required in five (33%) patients, of which three (20%) were due to the previously reported DLTs leading to permanent study discontinuation.

### Pharmacokinetics

**Fig. 1:**
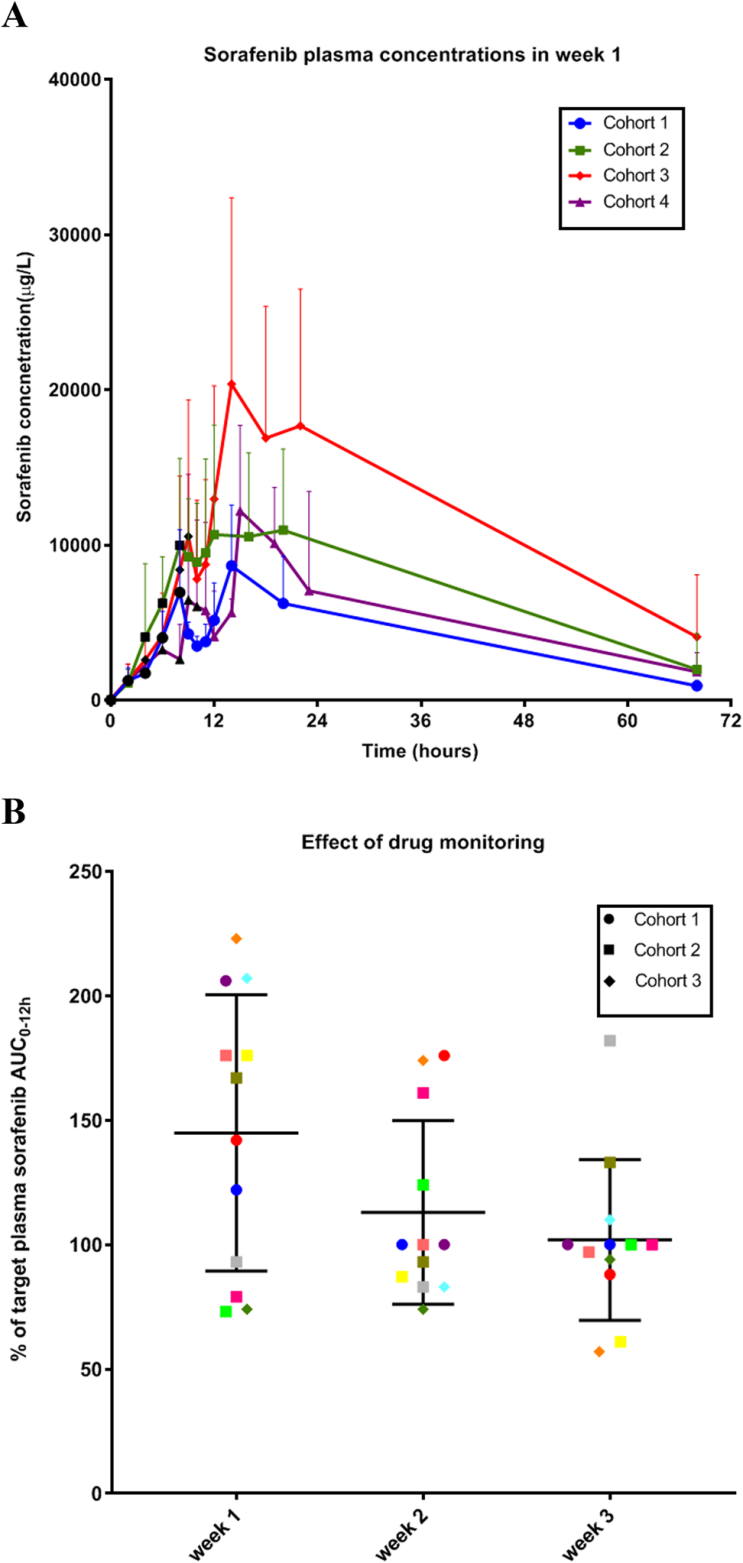
**a** High-dose, pulsatile sorafenib plasma concentrations (mean ± SD) in week 1 using a standard fixed dose for each cohort (*N* = 17, black dots are time points of sorafenib ingestion) and **b** shows the effect of drug monitoring (*N* = 12, each colored symbol represents an individual patient followed in weeks 1–3)

Plasma samples for pharmacokinetic analyses were available from all 17 included patients and results are summarized in Table 3. Mean exposure increased with higher sorafenib doses up to 2400 mg per week, after which no additional increases were seen. This is possibly related to a saturation of uptake in the gastrointestinal tract. The increases were similar for AUC(0–12 h) as well as *C*_max_ throughout the escalating exposure cohorts. In the current phase I study, highest exposure levels were up to 204 mg/L/h (SD ± 113 mg/L/h) with a *C*_max_ up to 21.0 mg/L (SD ± 11 mg/L), reached at median 7 h after ingestion (range 1–12 h). The plasma concentration time curves of sorafenib showed a biphasic pattern, which has been described previously, and is most likely caused by biliary excretion and the enterohepatic cycle (Fig. 1). No accumulation of sorafenib was seen a week after each ingestion. The major metabolite of sorafenib, sorafenib N-oxide, comprised approximately 4% of the parent drug and showed similar *C*_max_ and AUC(0–12 h) patterns compared to sorafenib (Supplementary Figure S1).

**Table 3:** Pharmacokinetic results for high-dose, pulsatile sorafenib

Cohort	No. of patients	Target AUC(0–12 h) (mg/L/h)	Fixed dose week 1 (mg)	PK-guided dose week 3 (mg), mean (± SD)	Observed AUC(0–12 h) week 1 (mg/L/h), mean (± SD)	Observed AUC(0–12 h) week 3 (mg/L/h), mean (± SD)	*C*_max_ week 1 (μg/L), mean (± SD)	*C*_max_ week 3 (µg/L), mean (± SD)
1	3	25–50	1000	533 (± 306)	78 (± 22)	31 (± 13)	9089 (± 3281)	4385 (± 1312)
2	8	75–100	2000	1867 (± 993)	125 (± 59)	102 (± 49)	13,696 (± 5866)	11,098 (± 6115)
3	3	100–125	2400	2333 (± 219)	204 (± 113)	96 (± 40)	20,829 (± 11,234)	13,280 (± 5310)
4	3	125–150	2800	NA*	120 (± 13)	NA*	15,200 (± 1266)	NA*

*NA not applicable

At start of treatment, with a pulsatile fixed high-dose, sorafenib exposure showed large interpatient variability with a mean difference between observed and target AUC(0–12 h) of 45% (SD ± 56%) in week 1 (Fig. 1). Personalized dose titration resulted in a mean difference between observed and target AUC(0–12 h) of 2% (SD ± 32%) in week 3. The difference between week 1 and week 3 showed a trend towards an improved prediction of exposure (*P* = 0.06).

**Fig. 2:**
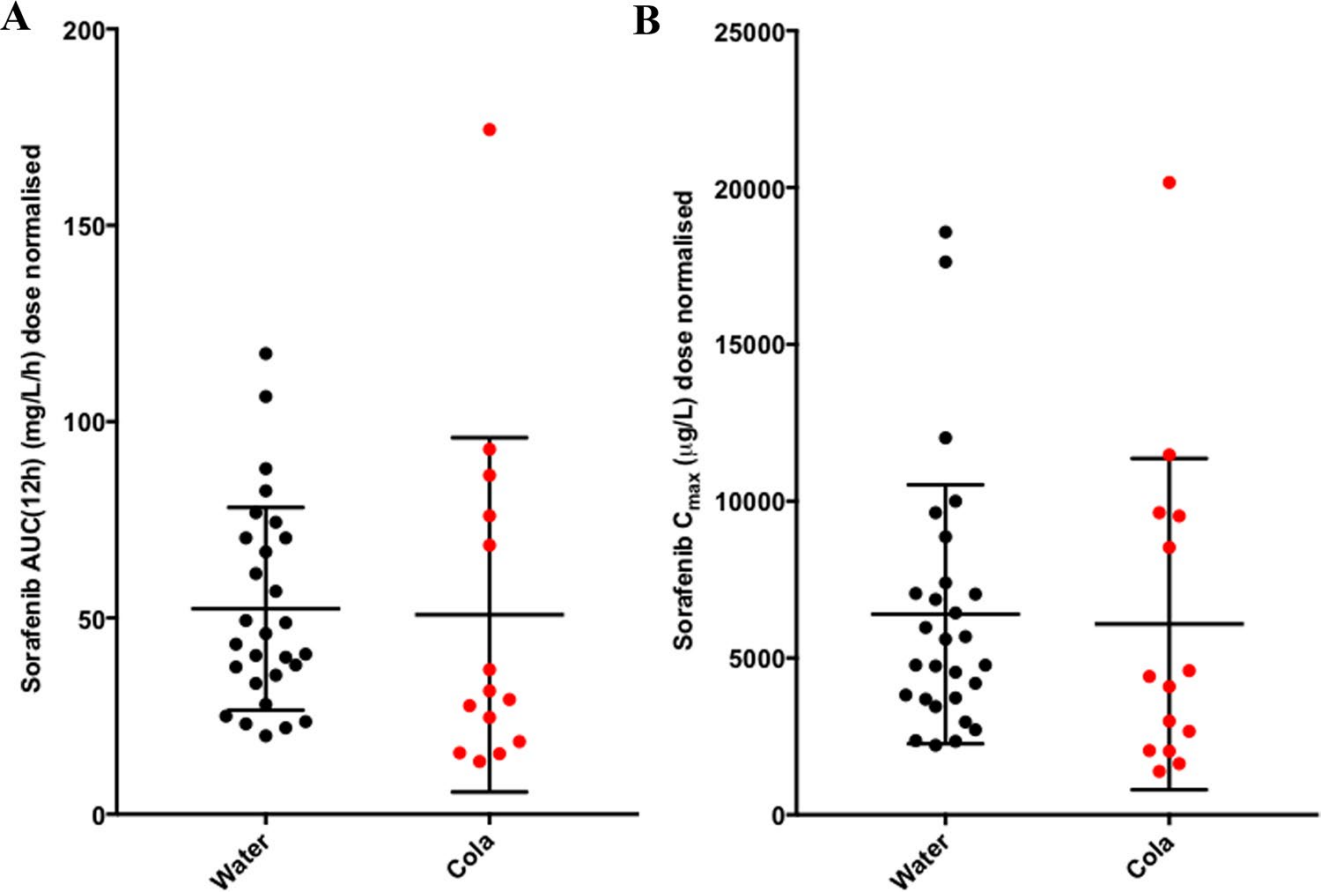
Effect of cola on sorafenib absorption. Cola did not affect sorafenib AUC(0-12 h) (**a**) or *C*_max_ (**b**). Black and red dots represent sorafenib dissolved in water and cola, respectively

All patients in this phase I study used proton pump inhibitors (PPI) for various reasons, which could decrease sorafenib absorption as a result of an increasing gastric pH. To lower the pH, sorafenib was dissolved in cola, but this did not lead to an increase in sorafenib AUC(0–12 h) or *C*_max_ compared to patients treated with sorafenib dissolved in water (*P* = 0.24 and 0.33, respectively) (Fig. 2).

### Treatment efficacy

Thirteen patients were evaluable for response. As best response, two patients (15%) had stable disease: a patient with cholangiocarcinoma for a duration of 3 months and treated at the target exposure level of 75–100 mg/L/h (sorafenib dose was 2400 mg/week and observed AUC(0–12 h) was 100 mg/L/h in week 3) and another patient with hepatocellular carcinoma for a duration of 5.5 months, who was treated at the target exposure level of 100–125 mg/L/h (sorafenib dose was 4800 mg/week and observed AUC(0–12 h) was 94 mg/L/h in week 3). The latter patient was previously progressive during treatment with sorafenib at the standard continuously dosed schedule. Eleven patients (85%) had progressive disease. No complete or partial responses were observed.

## Discussion

In this phase 1 study, a high-dose, intermittent sorafenib schedule was investigated and dose escalation was performed according to a novel concept, i.e. based on escalating sorafenib plasma AUC(0–12 h) levels, instead of conventional dose escalating cohorts. The aim was to reach the highest tolerable plasma sorafenib peak concentration supposed necessary for the highest intratumoral concentration to improve blockade of tumor kinase targets. With the standard continuous sorafenib schedule of 400 mg twice daily, mean sorafenib exposure varied from 21.8–107 mg/L/h on day 1 and 47.8–71.7 mg/L/h at steady state, while mean *C*_max_ values ranged from 2.9–3–4 mg/L on day 1 to 5.4–9.4 mg/L at steady state, which was reached approximately 3 h after ingestion (range 0–24 h) [8, 26]. High-dose pulsatile sorafenib resulted in a *C*_max_ up to 21.0 mg/L (SD ± 11 mg/L), i.e. approximately sevenfold and twofold higher in comparison to a single dose and continuous standard dosing, respectively.

A few other protein kinase inhibitors have been investigated in a high-dose, pulsatile schedule. High-dose erlotinib administered at a dose of 2000 mg per week in NSCLC patients was well tolerated and resulted in a mean overall survival (OS) of 9.5 months [27]. Another phase II study investigated high-dose erlotinib 450 mg every 3 days or the EGFR inhibitor gefitinib 1000 mg every 4 days in patients with known EGFR mutations and disease progression after treatment with conventional dose erlotinib or gefitinib [28]. Treatment was well tolerated and resulted in a median PFS of 6 months in both groups and response rates of 15 and 21%, respectively. In addition, a phase I study escalating the HER2 inhibitor lapatinib in heavily pre-treated patients with HER2-positive breast cancer to 7000 mg on days 1–5 of repeating 14-day cycles showed an objective response rate in 15% of the patients [29]. We recently investigated the multikinase inhibitor sunitinib in a high-dose, pulsatile phase I study in more than 70 heavily pre-treated patients with advanced solid malignancies [13]. The study showed that a high-pulsatile schedule of 14 times the conventional dose of sunitinib was well tolerated and led to an 18-fold higher *C*_max_. In addition, the drug showed promising preliminary efficacy with clinical benefit in 63% of the patients, including a PFS of ≥ 5 months in 30% of the patients and is currently being investigated in two phase II trials (NCT03909724 and NCT03025893). Unfortunately, high-dose, pulsatile sorafenib exposure did not achieve sufficiently increased peak concentration levels, which were considered necessary for improved efficacy. With only ~3.5 times its conventional dose tolerated in a pulsatile schedule, a peak concentration of only 2 times the standard C_max_ was attained in this study. Drug exposure is influenced by its absorption in the stomach which is dependent of factors such as diet and pH. The patients ingested sorafenib with a moderate fat meal and multiple divided doses of 200–400 mg every 2 h, to maximize absorption. This resulted in increased exposure up to sorafenib doses of 2400 once per week, which is 3 times higher than the previously reported saturation> 800 mg sorafenib per day using the standard continuous schedule [15]. Regarding the stomach pH, there have been contradicting results on the effects of PPI on sorafenib absorption, varying from no effects to one-third reduction of sorafenib absorption [17, 18]. We observed that an acidic beverage such as cola did not improve sorafenib exposure in patients using a PPI. Previously, it was shown that erlotinib bioavailability did improve by cola intake in patients using omeprazole [20]. However, erlotinib is poorly soluble in water, while the maximal aqueous solubility of 0.4 mg/mL occurs at pH ~2.0 [19]. Thus, the absolute differences in solubility for erlotinib dependent on stomach pH are much higher than for sorafenib, which ranges from 0.013 mg/100 mL at pH 4.5 to 0.034 mg/100 mL at pH 1.0 [17–19].

In this phase 1 study, dose escalation was performed in exposure escalation cohorts, instead of conventional dose escalation cohorts, because sorafenib exposure has large interpatient variability using a fixed dose [8]. Drug monitoring of sorafenib, with a maximum of two dose adjustments, resulted in a difference of 2% between observed and target AUC(0–12 h) compared to 45% at the start of treatment using a fixed dose. Although this was only borderline significant (*P* = 0.06) in this small patient group, the feasibility of sorafenib drug monitoring to achieve a target exposure supports this strategy to improve controlled drug exposure. Further research is necessary to investigate whether dose titration based on exposure will lead to improved efficacy.

Unfortunately, we observed considerable toxicity in this phase 1 study with high-dose, intermittent sorafenib. Grade 5 biliary tract perforation was observed in a patient treated at the target exposure level of 75–100 mg/L/h and grade 3 duodenal perforation and grade 5 multiorgan failure in two separate patients treated at target exposure level of 100–125 mg/L/h. We found that ≥ grade 3 toxicity was associated with increased ingested sorafenib dose (doses ≥ 2800 mg/week), but not with plasma sorafenib AUC(0-12 h). Because sorafenib is a multikinase inhibitor, including inhibition of angiogenesis, perforation of the gastrointestinal tract is a well-known side effect, but occurs at a low incidence in < 1% of the patients treated at the standard continuous schedule [30, 31]. We therefore took precautions to prevent perforations by excluding patients with previous radiotherapy of the thoracic/bowel region, as other studies showed this was a risk factor for gastrointestinal perforation in combination with VEGF inhibitors [13, 32, 33]. Several phase I studies have investigated the safety and pharmacokinetics of standard dose sorafenib and the most important DLTs were skin toxicity, diarrhea and fatigue [23]. However, this was the first study to investigate the safety and pharmacokinetics of sorafenib in a high-dose, pulsatile schedule, which showed a different DLT profile. The incidence of perforation was 13%. The frequency and type of serious toxicities (perforation and multiorgan failure) observed at only ~3.5 times the conventional sorafenib dose in a weekly schedule, were unexpected and reason for preliminary study termination. This was in contrast to the example of high-dose, pulsatile sunitinib, which is also an anti-angiogenic drug and showed tolerability comparable to daily administration up to 14 times the conventional dose [13]. A possible explanation may be the enterohepatic circulation of sorafenib. This encompasses the hepatobiliary excretion of sorafenib and a second round of exposure of the intestinal tract to sorafenib with subsequent partial reabsorption. Enterohepatic circulation has been observed in animal models for sorafenib, but not for sunitinib, and is also an explanation for the biphasic plasma concentration time curve typical for sorafenib and observed in the current study [16, 34–37]. In addition, in this pulsatile weekly regimen, sorafenib doses were divided over consecutive 2-h dose administrations. For instance, a 3600 mg dose was administered as 9 consecutive doses of 400 mg, which was done to circumvent the saturable absorption of sorafenib. As a result, local drug concentrations in the GI tract were high during prolonged periods of time and may have caused local anti-angiogenic effects in the GI tract, inducing DLT. The sorafenib dose administered is a better measure of local GI tract sorafenib exposure as opposed to the observed plasma concentrations of sorafenib. This also explains why adverse events were not associated with sorafenib exposure, but rather with sorafenib dose. In contrast to the observed DLT in this study, we only observed mild diarrhea (< grade 3) in 27% of the patients. This may indicate that the anti-angiogenic effects of high-dose pulsatile sorafenib were more pronounced than other cytotoxic effects responsible for diarrhea, such as inhibition of the MAPK signaling pathway that can lead to increased chloride secretion by the normal GI mucosa and subsequent secretory diarrhea [38, 39]. While the pulsatile regimen was targeting high exposure (irrespective of dose), the study was discontinued early due to the occurrence of unexpected, serious DLT (despite relatively low plasma exposure). These results may help to guide the future selection of protein kinase inhibitors suitable for an alternative, pulsatile, high-dosing schedule.

In conclusion, we have shown the feasibility of drug monitoring to achieve exposure-based treatment cohorts for high-dose, pulsatile sorafenib. Unfortunately, potentially effective high peak concentrations could not be reached due to early toxicity at already lower concentrations than anticipated. Dose escalation above the exposure cohort of 125–150 mg/L/h was impossible, so the potential benefit of this alternative approach could not be investigated further.

## Electronic supplementary material

Below is the link to the electronic supplementary material.

Supplementary file1 Sorafenib versus sorafenib N-oxide AUC(0–12 h) (a) and Cmax (b) (TIF 1139 kb)
