# Supplementary figures and images for: Sorafenib administered using a high-dose, pulsatile regimen in patients with advanced solid malignancies: a phase I exposure escalation study
Source: Cancer Chemother Pharmacol. 2020 Apr 9;85(5):931–40. doi: 10.1007/s00280-020-04065-5 (PMC7188706; doi:10.1007/s00280-020-04065-5)

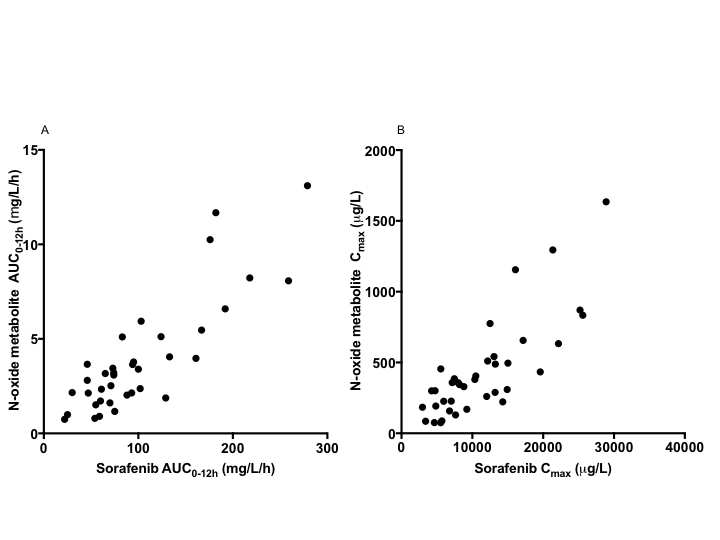

Supplement: Supplementary file 1 — Supplementary file1 Sorafenib versus sorafenib N-oxide AUC(0–12 h) (a) and Cmax (b) (TIF 1139 kb) [file 280_2020_4065_MOESM1_ESM.tif]
